# Supplementary material for: The Optical Response of a Mediterranean Shrubland to Climate Change: Hyperspectral Reflectance Measurements during Spring
Source: Plants (Basel). 2022 Feb 12;11(4):505. doi: 10.3390/plants11040505 (PMC8874438; doi:10.3390/plants11040505)
Supplement: Supplementary file 1 [file plants-11-00505-s001.zip › plants-1585141-supplementary.pdf]

plants

MDPI

Article

The Optical Response of a Mediterranean Shrubland to Climate Change: Hyperspectral Reflectance Measurements during Spring

Jean-Philippe Mevy [1,*], Charlotte Biryol [1], Marine Boiteau-Barral [1] and Franco Miglietta [2,3]

1 IMBE-UMR CNRS 7263/IRD 237, Aix-Marseille Université, 13331 Marseille, France charlotte.biryol@imbe.fr (C.B.); marine.boiteau-barral@etu.univ-amu.fr (M.B.-B.)
2 Institute of Biometeorology, National Research Council (CNR- IBIMET), Via Caproni 8, 50145 Firenze, Italy; f.miglietta@gmail.com
3 IMèRA, Institut d'Etudes Avancées de l'Université Aix-Marseille, 2 Place Le Verrier, 13004 Marseille, France
* Correspondence: jean-philippe.mevy@univ-amu.fr; Tel.: +33-0413550766

Supplementary Materials

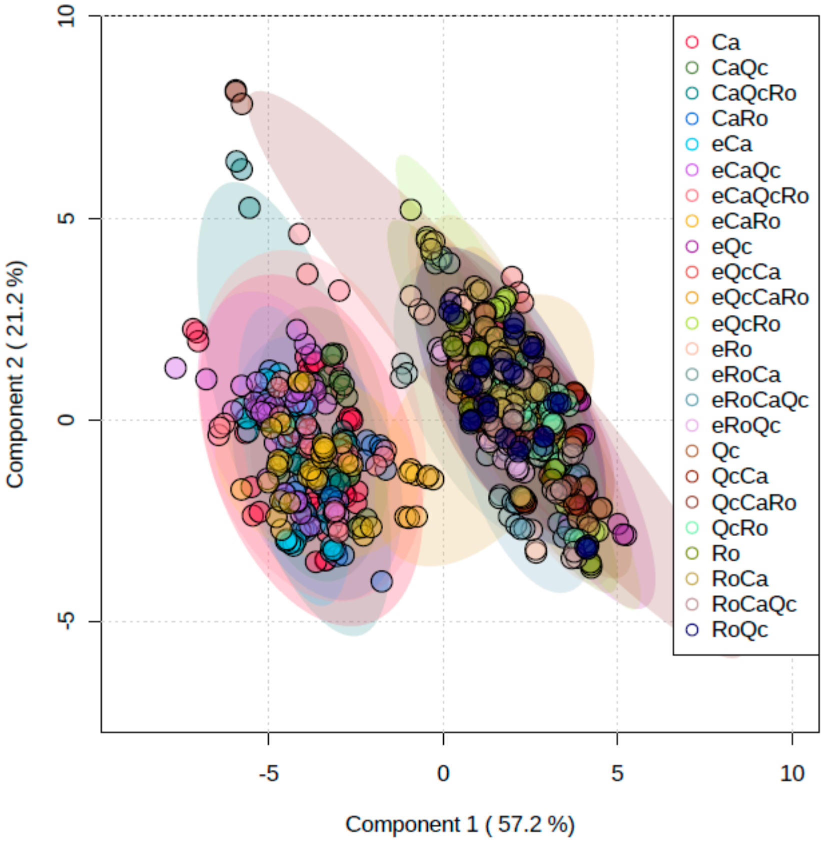

Figure S1. Partial Least Squares Discriminant Analysis (PLS-DA) of Cistus albidus (Ca) Rosmarinus officinalis (Ro) and Quercus coccifera (Qc) in monospecific (Ca; Qc; Ro), bi-specific (CaQc; CaRo; QcCa; QcRo; RoQc) and tri-specific (CaQcRo; QcCaRo; RoCaQc) stands. Plan of individuals on axes 1 and 2. In plurispecific assemblages the first 2 letters correspond to the species from which the measurements were carried out in the assemblage. Exclusion device (e), the others are controls.

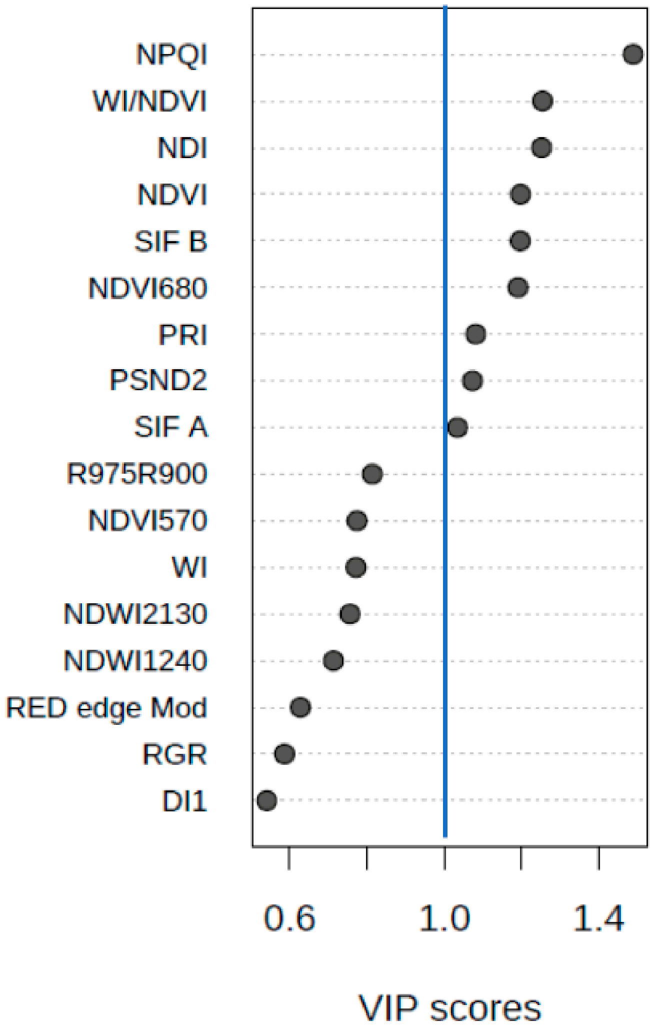

Figure S2. The important vegetation indices identified by PLS-DA with a cut-of at 1.
